# Haploinsufficiencies of *FOXF1*, *FOXC2* and *FOXL1* genes originated from deleted 16q24.1q24.2 fragment related with alveolar capillary dysplasia with misalignment of pulmonary veins and lymphedema-distichiasis syndrome: relationship to phenotype

**DOI:** 10.1186/s13039-022-00627-9

**Published:** 2022-11-03

**Authors:** Xuezhen Wang, Lili Guo, Bei Zhang, Jiebin Wu, Yu Sun, Huimin Tao, Jing Sha, Jingfang Zhai, Min Liu

**Affiliations:** 1 Graduate School of Bengbu Medical College, Donghai Avenue No. 2600, Bengbu, 233000 Anhui China; 2 Department of Prenatal Diagnosis Medical Center, Xuzhou Central Hospital, No. 199 South Jiefang Road, Xuzhou, 221009 Jiangsu China; 3 Graduate School of Xuzhou Medical University, Jiangsu, 221000 Xuzhou China; 4 Department of Obstetrics, Fengxian People’s Hospital, Feng Xian Renmin West Road No.51, Xuzhou, 221700 Jiangsu China

**Keywords:** ACDMPV, LDS, Haploinsufficiencies of *FOXF1*, *FOXC2* and *FOXL1* genes, Multiple-system structural malformations, Prenatal diagnosis

## Abstract

**Objective:**

We describe a fetus with a 2.12-Mb terminal deleted fragment in 16q associated with alveolar capillary dysplasia with misalignment of pulmonary veins (ACDMPV) and lymphedema-distichiasis syndrome (LDS) and intend to provide a comprehensive prenatal management strategy for the fetuses with ACDMPV and LDS through reviewing other similar published studies.

**Methods:**

The fetus presented a series of diverse structural malformations including congenital cardiovascular, genitourinary and gastro-intestinal anomalies in ultrasound at 23 + 5 weeks of gestation (GA). Amniocentesis was conducted for karyotype analysis and copy number variation sequencing (CNV-seq) after informed consent.

**Results:**

The fetal karyotype was 46,XX, however the result of CNV-seq showed an approximately 2.12-Mb deletion in 16q24.1q24.2 (85220000-87340000) × 1 indicating pathogenicity.

**Conclusion:**

Genomic testing should be recommend as a first line diagnostic tool for suspected ACDMPV and/or LDS or other genetic syndromes for the fetuses with structural abnormalities in clinical practice.

## Background

ACDMPV (OMIM 265380) is a rare and deadly disorder characterized by severe respiratory distress and cyanosis with the incidence of 1/100,000 [1]. In addition, about 50 to 75 percent of affected newborns have multiple-system abnormalities such as hypoplastic left heart syndrome (HLHS) and intestinal malrotation [2]. In approximately 80–90% of ACDMPV cases, heterozygous single nucleotide variants (SNVs) or copy number variant (CNV) deletions involving forkhead box F1 (*FOXF1*, OMIM 601089) in chromosome 16q24.1 have been found [3, 4]. In this report, we describe a fetus featured by a series of diverse structural malformations. Meanwhile, CNV-seq revealed a deleted region in 16q24.1q24.2 related with ACDMPV [5] and LDS [6]. Both ACDMPV and LDS (OMIM 153400) are rarely reported in adults simultaneously in practice because of nearly 100% mortality of the cases with ACDMPV in the newborn period [7]. However, the severity of isolated LDS associated with pathogenetic forkhead box C2 (*FOXC2*, OMIM 602402) is variable and cannot be predicted, among which the majority have been found in late childhood or adolescence with classical lymphatic abnormalities [8] and the minority in fetuses with nuchal translucency thickness [9–11]. Furthermore, we compare the features of our fetus with the reported cases related with 16q24.1q24.2 microdeletion syndromes. We aim to provide a comprehensive prenatal management strategy for the fetuses with ACDMPV and LDS.

## Materials and methods

### Case presentation

**Fig. 1:**
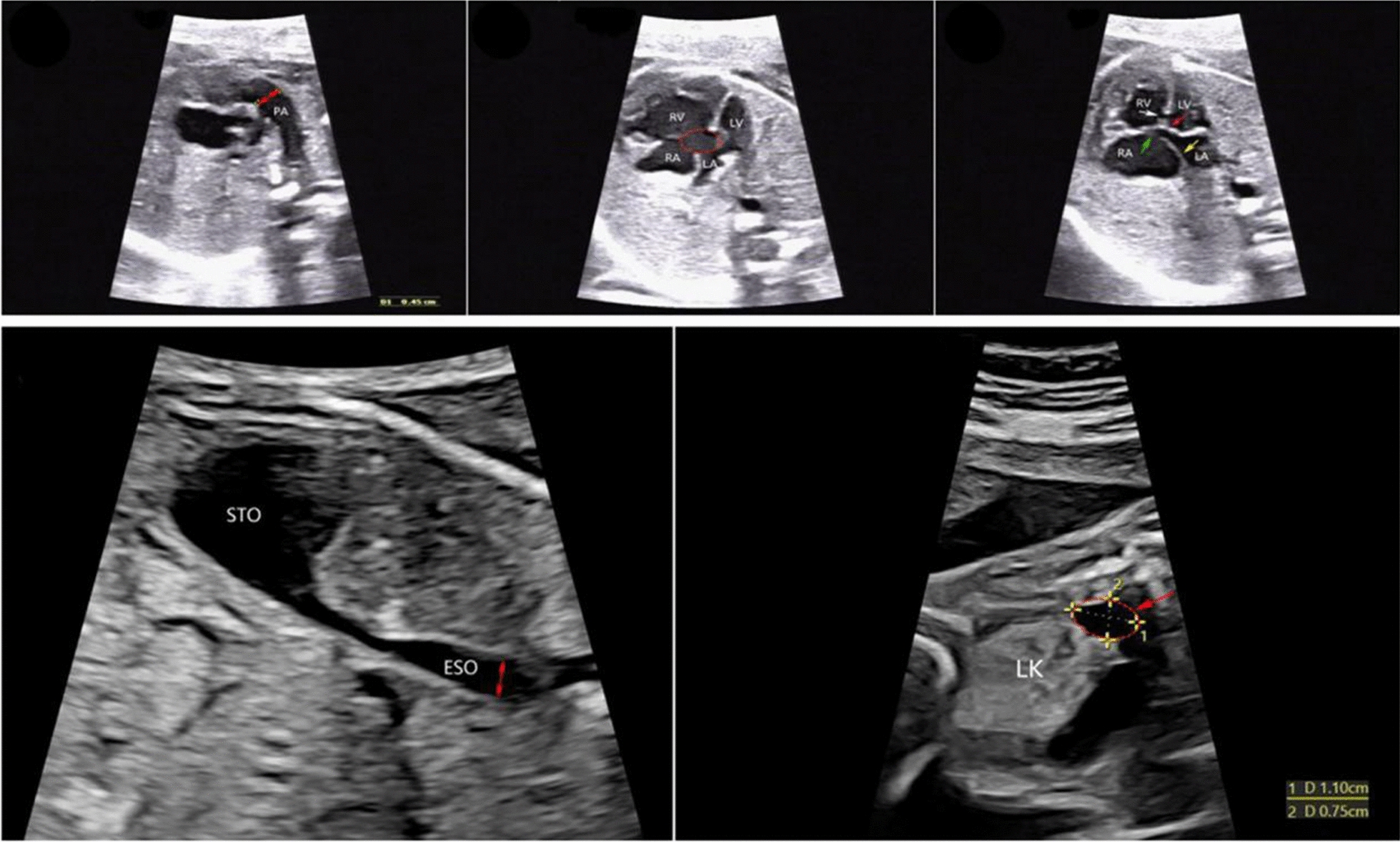
Fetal ultrasound at 23 + 5 weeks gestation showed **a** pulmonary artery (PA) dilatation with an internal diameter of about 4.5 mm; **b** complete atrioventricular septal defect manifestation during diastole; **c** common atrioventricular valve (red arrow), foramen ovale closure (yellow arrow), atrial septal defect with the width of 2.2 mm (green arrow), ventricular septal defect with the width of 2.6 mm (white arrow) and right heart enlargement manifestations during systole; **d** dilatation of the stomach measuring about 32 × 13 mm and esophageal dilation with the widest internal diameter of 9 mm (considering pyloric obstruction); **e** a 11 × 7.5 mm hypodense mass in the upper pole of the left kidney. Abbreviation: ESO, esophagus; LA, left atrium; LK left kidney; LV, left ventricle; PA, pulmonary artery; RA, right atrium; RV, right ventricle; STO, stomach

**Fig. 2:**
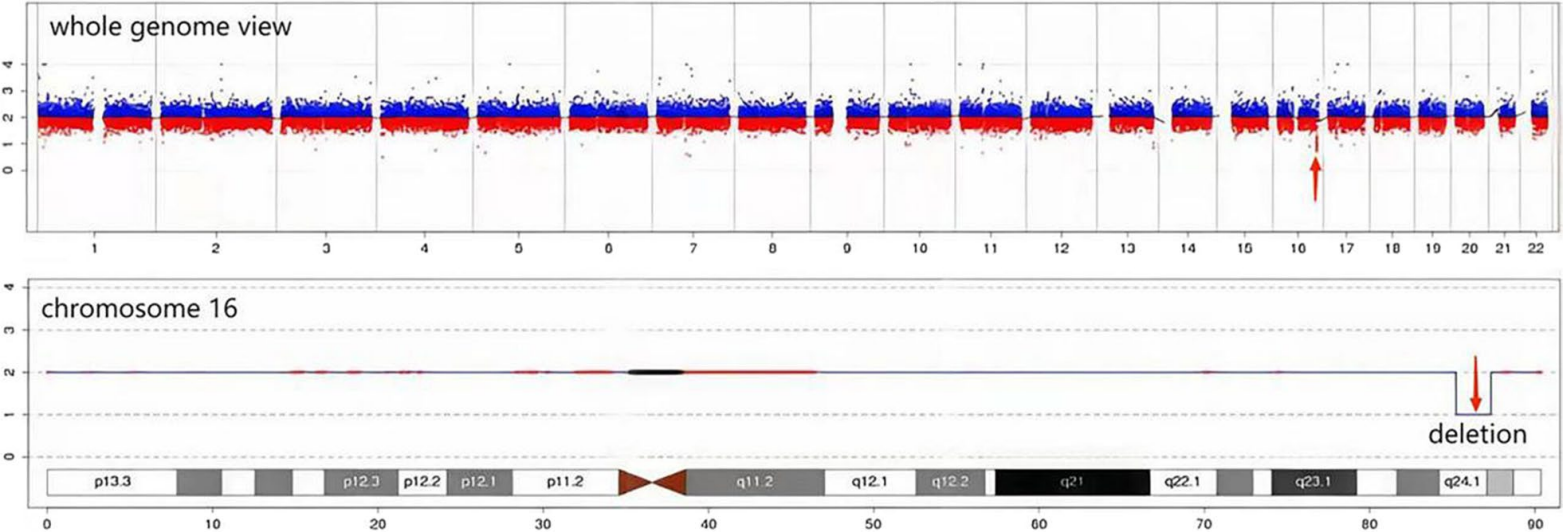
The CNV-seq result of fetus showed a 2.12-Mb deletion in 16q24.1q24.2 (85220000-87340000)

A 28-year-old healthy multigravida woman resorted to prenatal diagnosis medical center of Xuzhou Central Hospital due to abnormal ultrasound results. She had no history of adverse pregnancy and drug usage, and the couple were non-consanguineous. The family has a healthy child. There were not family histories with any serious disorders. Prenatal ultrasound at 23 + 5 weeks of GA showed the following presentations of Fig. 1: (a) pulmonary artery (PA) dilatation; (b) complete atrioventricular septal defect (AVSD); (c) common atrioventricular valve (CAV), foramen ovale closure (FOC), atrial septal defect (ASD), ventricular septal defect (VSD) and right heart enlargement; (d) dilatation of the stomach, esophageal dilation (considering pyloric obstruction); (e) a hypodense mass in the upper pole of the left kidney on December 23, 2022. Amniotic fluid was collected for karyotype analysis and CNV-seq after informed consent. Although the fetal karyotype was 46,XX, the result of CNV-seq showed that there was an approximately 2.12-Mb pathogenetic deletion in 16q24.1q24.2 (85220000-87340000) × 1 (Fig. 2) which was confirmed to be de novo after CNV-seq results of the couple were verified. Finally after receiving sufficient genetic counseling, the couple provided informed consent and chose to terminate the pregnancy. This study was approved by Xuzhou Central Hospital Ethics Committee (No. XZXY-LK-20210812-019).

### Methods

Chromosome analysis was performed on G-band metaphases from amniotic fluid sample according to the laboratory’s standard protocols.The following entire operation process of CNV-seq included extracting uncultured genomic DNA from the sample, constructing DNA libraries, massively sequencing in parallel and conducting the raw sequencing reads following the corresponding operating regulations [12]. Finally, the results of data were assessed according to standards and guidelines of American College of Medical Genetics [13].

## Discussion

**Fig. 3:**
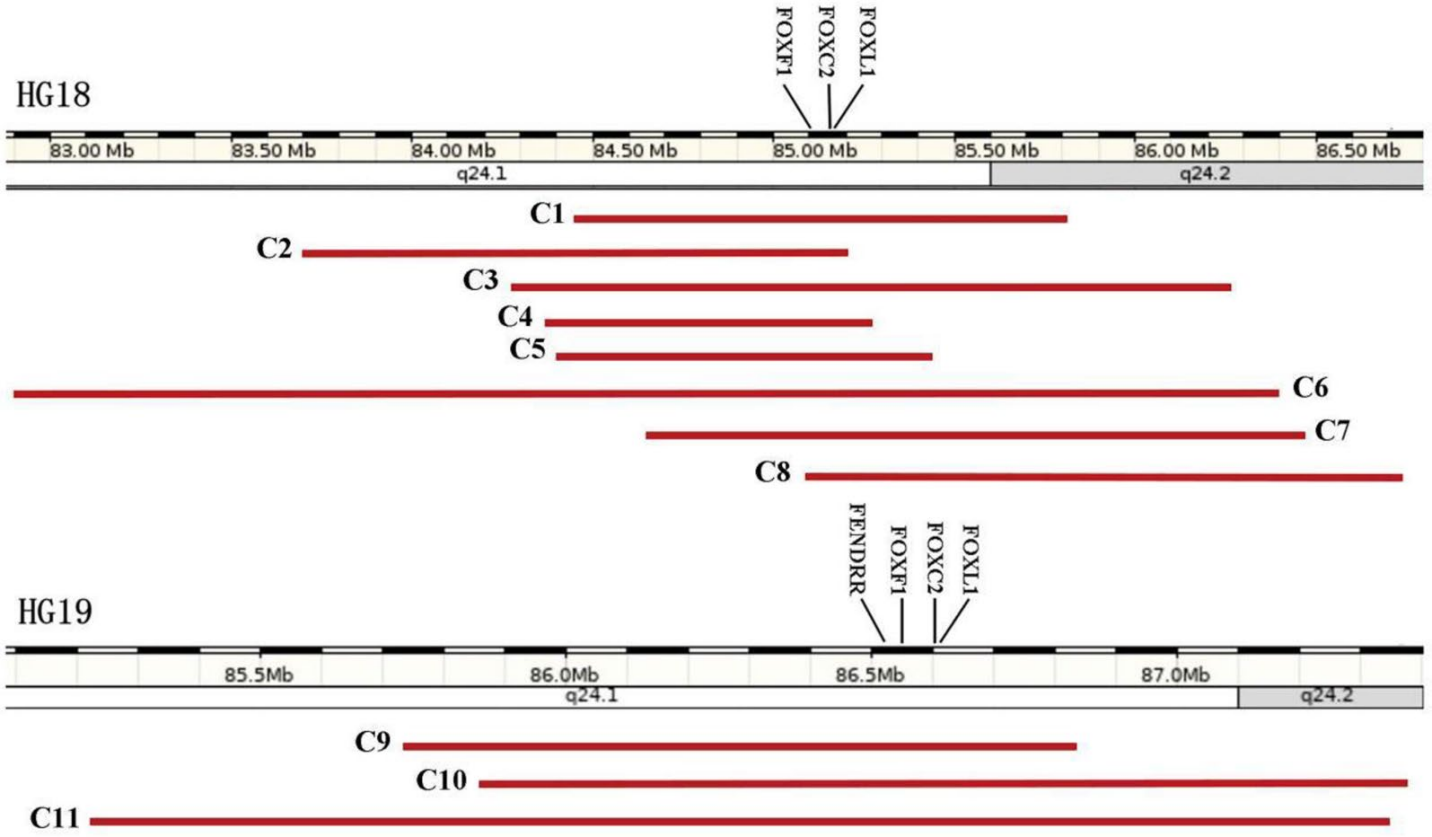
Schematic representation of the genomic region harboring *FOXF1*, *FOXC2*, and *FOXL1* showed the extent and primary gene content of the regions deleted in 11 cases, according to different versions of the genome map from UCSC Genome Browser Home: **a** cases from C1 to C8 were plotted with HG18; **b** cases from 9 to 11 with HG19

ACDMPV and LDS have been confirmed to be related with the deleted 16q24.1q24.2 fragment until now [5, 14]. In this case, CNV-seq detection showed a 2.12-Mb deleted region in 16q24.1q24.2 containing the following definite pathogenetic genes: *FOXF1*, *FOXC2* and related regulatory genes including forkhead box L1 (*FOXL1*, OMIM 603252) and *FOXF1* adjacent non-coding developmental regulatory RNA (*FENDRR*). Combined with the abnormal results of multi-system malformations of the fetus such as congenital cardiac, lung, genitourinary and gastro-intestinal anomalies, the diagnosis of ACDMPV and LDS of the fetus was further defined. In addition to our fetus, Table 1 shows the other 10 cases with similar deleted fragment in the 16q24.1q24.2 region with complete information, and the sizes range from 0.9 to 3.5 Mb containing *FOXF1*, *FOXL1* and *FOXC2* genes, among which two fetuses were from de novo disease-causing variants of the above genes, four cases from maternal heredity, four cases from unknown origin, three females and seven males are enrolled from five literatures [3, 15–18]. And we present a figure visualizing the deleted regions of 11 cases harboring *FOXF1*, *FOXC2*, and *FOXL1* according to different versions of the genome map from UCSC Genome Browser Home: (a) cases from C1 to C8 were plotted with HG18; (b) cases from 9 to 11 with HG19 (Fig. 3). As is shown, the deleted sizes of 16q24.1q24.2 fragment are not proportional to the severity of phenotypes, and both cardiac and renal anomalies are the two major manifestations during the fetal period, while the phenotypes of our fetus are the most serious, showing the changes of cardio-pulmonary structure such as PA dilatation, HLHS, complete AVSD, CAV, FOC, ASD, VSD; the upper pyloric obstruction manifestations; a hypodense mass in the left kidney. However, the prime symptoms of neonates after birth are featured by respiratory, gastro-intestinal and genitourinary manifestations. Moreover, the gestational ages of delivery range from 22 to 39 + 1 weeks, among which three couples opted to terminate the pregnancies at second trimester of pregnancy and all of them died of respiratory diseases and their lifespans ranged from 16 h to 40 days. Therefore, early recognition of ACDMPV and LDS is essential in clinical practice.

**Table 1:** Features of patients with 16q24.1q24.2 deletion harboring FOXF1, FOXL1 and FOXC2

Cases	1	2	3	4	5	6	7	8	9	10	11
References	15	16	16	16	16	16	16	17	18	3	Our case
Genome coordinates (hg18/ hg19)	chr16:84447762-85815086	chr16:83705765-85204004	chr16:84275154-86275754	chr16:84374208-85277007	chr16:84402571-85435712	chr16:82908199-86405076	chr16:84648160-86478255	chr16:85108709-86720212	chr16:85728812-86831579	chr16:85863000-87370500	chr16:85220000-87340000
Karyotype	NA	NA	NA	NA	NA	NA	NA	–	–	–	–
Deletions (del) [Mb]	1.37	1.5	2.0	0.9	1.0	3.5	1.8	1.57	1.1	1.45	2.12
Female/Male	Male	Female	Male	Male	Female	Male	Female	Male	Male	Male	Female
Inheritance	De novo	Maternal	NA	Maternal	Maternal	De novo	Maternal	NA	NA	NA	De novo
Other pathogenic genes	IRF8; FOXL1	FOXL1	FOXL1	FOXL1	FOXL1	FOXL1	FOXL1	IRF8; FOXL1	IRF8; FOXL1; COX4I1	IRF8; FOXL1; FENDRR	FOXL1; IRF8; COX4I1; FENDRR
Prenatal fingdings	BH; PE; PHD; HLHS	NA	NA	NA	NA	NA	NA	PH; partial AVC defect; BH	Cystic hygroma; fetal hydrops; SUA	PHD; omphalocele; hydronephrosis and VSD	Widened PA; AVSD; CAV; FOC; mass in kidney; SD; ED
Delivery GA. (W)	37	28	22	38	37	NA	NA	26	22	39 + 1	23 + 6
Birth Wt. (g)	NA	1091	NA	2900	NA	NA	3676	592.4	NA	2920	NA
Respiratory findings	ACD/MPV	ACD/MPV; PL	–	ACD/MPV; ECMO dependent	ACD/MPV; LP; hypoxemia; ECMO dependent	ECMO dependent	ACD/MPV	ACD/MPV	–	ACD/MPV	NA
LDS	–	–	–	–	–	–	–	–	–	–	–
Cardiac findings	HLHS; PVA; small main PA; VSD; ASD; PDA; PLSVC; CP	PDA	HLHS	TOF; PDA; PPHN	HLHS	IAA; dilated PA; large PDA; small LV; PH	PDA; PPHN	Partial AVC malformation; Small PA	–	PPHN; ASD; VSD	NA
Genitourinary findings	Hydronephrosis; hypospadias	–	Dilated renal pelvices	BH	Mild uretero-pelvic caliectasis	Bilateral renal pelviectasis	–	Bilateral dilatation of the PS with bilateral US	–	–	NA
Gastro-intestinal findings	IM; ectopic cecum and appendix	EA; TSF; ectopic anus	–	DA; AP; imperforate anus	–	–	Adhesions between bowel loops、 duodenum and gallbladder	AP; duodenal dilatation proximal to the pancreas	–	Lack of peristalsis	NA
Other findings	HP; flat nasal bridge; HM; decreased muscle tone	SUA	–	SUA	T11 butterfly vertebra; cleft lip; cleft palate; brachycephaly; SUA	Posterior rib fusions: 10/11 (right side), 9/10 and 11/12 (left side)	–	Intrauterine infection	Low set ears and soft tissue edema of the neck	Coagulopathy; metabolic acidosis	NA
LS	3 days	1 days	/	40 days	15 days	18 days	25 days	16 h	/	13 days	/

*AP* annular pancreas, *ASD* atrial septal defect, *AVC* atrio-ventricular canal defect, *AVSD* atrioventricular septal defect, *BH* bilateral hydronephrosis, *CAV* common atrioventricular valve, *CP* cor pulmonale, *DA* duodenal atresia, *EA* esophageal atresia, *ECMO* extracorporeal membrane oxygenation, *ED* esophageal dilation, *FOC* foramen ovale closure, *GA* gestation, *HLHS* hypoplastic left heart syndrome, *HM* holosystolic murmur, *HP* hypertelorism, *IAA* interrupted aortic arch, *IM* intestinal malrotation, *LP* left pneumothorax, *LS* lifespan, *LV* left ventricle, *NA* not available, *PA* pulmonary artery, *PDA* patent ductus arteriosus, *PE* pleural effusion, *PH* pulmonary hypertension, *PHD* polyhydramnios, *PL* pulmonary lymphangiectasia, *PLSVC* persistent left superior vena Cava, *PPHN* persistent pulmonary hypertension of the newborn, *PS* pelvocaliceal system, *PVA* pulmonary valve atresia, *SD* dilatation of the stomach, *SUA* single umbilical artery, *TOF* tetralogy of Fallot, *TSF* tracheae-sophageal fistula, *US* ureteral stenosis, *VSD* ventricular septal defect, “–” normal

The CNV-seq result of our fetus indicated a 2.12-Mb deleted fragment in 16q24.1q24.2 (Fig. 2) including the FOX family of transcription factors (*FOXF1*, *FOXL1* and *FOXC2*), *FENDRR*, and *FOXF1* corresponding enhancer region. The FOX transcription factors play critical roles in the process of cellular proliferation, differentiation [19, 20]. *FOXF1* involves in development of pulmonary alveoli, capillaries and embryonic development of organs associated with airways, gastrointestinal tract and urinary tract in diverse-type cells including capillary endothelial cells, fibroblasts, and peribronchial smooth muscle cells [21, 22]. In epithelial cells of the peripheral lung mesenchyme, sonic hedgehog (SHH) signaling pathway mediated by *FOXF1* is one of the key pathways regulating formation. Moreover, the interactions between *FOXF1*-SHH and semaphorins-neuropilin or vascular endothelial growth factor/vascular endothelial growth factor receptor 2 (VEGF/VEGFR2) signaling may result in structural abnormalities of multiple systems, especially the lung, cardiovascular, gastrointestinal and urinary systems [22]. Hence, the haploinsufficiency of *FOXF1* gene is related with manifestations of lung, gastrointestinal and urinary tracts such as HLHS, duodenal atresia and distal ureteral dilatation [5, 16, 22] because of point disease-causing variant of *FOXF1* or CNV deletions overlapping *FOXF1* or the change of its upstream regulatory region located ~ 270 kb upstream to *FOXF1* gene (chr16:86178434-86238313, hg19) [4]. In addition, the genetic effects of *FOXF1* gene inactivation have been confirmed in *FOXF1*-deficient mice with severe alveolarization and angiogenesis defects, stenosis of esophageal and tracheal, lung repair defects, et al. [16, 23]. In our case, the fetus presenting similar multi-system clinical manifestations may be associated with the haploinsufficiency of *FOXF1*.

The deleted fragment in our fetus includes the other three genes—*FOXC2*, *FOXL1* and *FENDRR*. *FOXC2* is the key gene of LDS characterized by lymphedema of the limbs and double rows of eyelashes [14, 24], which is essential for lymphatic valve maintenance by regulating lymphatic endothelial cells junctional integrity and cellular quiescence [25]. *FOXC2* pathogenetic variant has been identified in cases with LDS to impair transcriptional activity and cell proliferation [26] through VEGF-C/VEGFR3 signaling pathway commonly correlated with primary lymphedema, lymphatic valve formation and other lymphatic malformations [27]. The *FOXC2*-inactiviation mice exhibited lymphatic abnormalities, VSD, interrupted aortic arch, et al. [28, 29]. In this report, although the characteristic phenotypes associated with LDS may be atypical in the fetal stage, CNV-seq detection confirms the diagnosis of LDS. Therefore, genetic detection should be recommended as a first-line diagnostic tool for the fetuses with suspected ACDMPV and/or LDS early during the fetal period [30]. In addition, the disease-causing variant of *FOXL1* gene is mainly related with gastrointestinal manifestations, as has been confirmed in mice with *FOXL1* gene knocked out [31]. Furthermore, *FENDRR* gene expression has been verified to be regulated both in cis and in trans by *FOXF1*, indicating that *FENDRR* involves in *FOXF1*-linked diseases including ACDMPV [32]. Therefore, we speculate that the present phenotypes of our fetus resulted from the deleted 16q24.1q24.2 fragment including *FOXF1*, *FOXC2*, *FOXL1* and *FENDRR*, and the severity might derive from the integration of multiple genes disease-causing variants of the above four genes. Our fetus has been confirmed with ACDMPV and LDS through CNV-seq detection.

In conclusion, this case supports the value of antenatal CNV-seq detection in multiple congenital abnormalities of the fetus. And genetic testing should now be recommend as a first-line diagnostic tool for suspected ACDMPV and/or LDS or other genetic syndromes for the fetuses with structural abnormalities in clinical practice, which may switch traditional histological examination of ACDMPV especially during the fetal period.

## Data Availability

The data and materials in the current study were available from the corresponding author upon reasonable request.

## Abbreviations

ACDMPV: Alveolar capillary dysplasia with misalignment of pulmonary veins
ASD: Atrial septal defect
AVSD: Atrioventricular septal defect
CAV: Common atrioventricular valve
CNV-seq: Copy number variation sequencing
*FENDRR*: *FOXF1* Adjacent non-coding developmental regulatory RNA
FOC: Foramen ovale closure
FOX: Forkhead-box
*FOXC2*: Forkhead box C2
*FOXF1*: Forkhead box F1
*FOXL1*: Forkhead box L1
GA: Gestation
LDS: Lymphedema-Distichiasis syndrome
PA: Pulmonary artery
SHH: Sonic hedgehog
VSD: Ventricular septal defect
VEGF/VEGFR2: Vascular endothelial growth factor/vascular endothelial growth factor receptor 2
